# Local management of the COVID-19 pandemic in Norway: a longitudinal interview study of municipality chief medical officers

**DOI:** 10.1080/02813432.2023.2301562

**Published:** 2024-02-07

**Authors:** Silje Rebekka Heltveit-Olsen, Lene Lunde, Anja Maria Brænd, Ivan Spehar, Sigurd Høye, Ingmarie Skoglund, Pär-Daniel Sundvall, Guro Haugen Fossum, Jørund Straand, Mette Bech Risør

**Affiliations:** a Department of General Practice, The Antibiotic Centre for Primary Care, Institute of Health and Society, University of Oslo, Oslo, Norway; b Department of Public Health Science, Institute of Health and Society, University of Oslo, Oslo, Norway; c Department of General Practice, General Practice Research Unit (AFE), Institute of Health and Society, University of Oslo, Oslo, Norway; d Department of Health Management and Health Economics, Institute of Health and Society, University of Oslo, Oslo, Norway; e Institute of Psychology, Oslo New University College, Oslo, Norway.;; f General Practice/Family Medicine, School of Public Health and Community Medicine, Institute of Medicine, Sahlgrenska Academy, University of Gothenburg, Gothenburg, Sweden.;; g Research, Education, Development and Innovation, Primary Health Care, Region Västra Götaland, Sweden.;; h Department of Public Health, The Research Unit for General Practice, University of Copenhagen, Copenhagen, Denmark; i The General Practice Research Unit, Department of Community Medicine, UiT The Arctic University of Norway, Tromsø, Norway

**Keywords:** COVID-19, qualitative research, public health, crisis management, municipality chief medical officers

## Abstract

**Objective:**

To explore the experiences and views of Norwegian Municipality Chief Medical Officers (MCMOs) on preparedness, collaboration, and organization during the COVID-19 pandemic to gain insight into local crisis management of value for future pandemic responses.

**Design:**

Longitudinal qualitative interview study. We conducted semi-structured digital interviews with nine MCMOs working in different municipalities in Norway from September to December 2020. Five MCMOs were re-interviewed from January to April 2021. We used thematic analysis to analyze the data.

**Results:**

Through the analysis, three major themes were identified in the material; 1) The view of preparedness changed from being low-priority and dormant to the desire to strengthen preparedness as a permanent measure; 2) The nature of the pandemic forced a change in internal and external communication and collaboration for the MCMOs towards direct dialogue, teamwork and digital networking; 3) The pandemic changed the role and position of the MCMO within the municipal organization. Although most MCMOs were given a leading role in the municipal pandemic response, some MCMOs experienced that they were not positioned to fully exercise their intended role. In our material, de-authorization of the MCMO role seemed to coincide with the increasing size and organizational complexity of the municipality.

**Conclusions:**

The Norwegian pandemic response and outcome have been regarded as successful internationally. Although the MCMOs managed to implement flexible and quick responses facilitated by teamwork, dialogue, and joint sensemaking, they also identified several challenges and shortcomings of the Norwegian pandemic preparedness requiring organizational and financial changes to sustain future health system resilience.

## KEY POINTS

- The Norwegian Infection Control Act gave comprehensive responsibility and authority for local COVID-19 pandemic management to the municipalities and the Municipality Chief Medical Officers (MCMOs).
- The MCMOs highlighted several challenges and shortcomings of the municipal crisis preparedness, of which lack of detailed organizational plans was the most prominent.
- Teamwork, digital networking and collective sensemaking seemed to enhance pandemic collaboration and resilience within and across municipalities.
- Most MCMOs gained a leading role at a higher organizational level within the municipality through the COVID-19 pandemic.
- To strengthen future crisis management, arrangements must be made, both organizationally and financially, for preparedness to remain on the agenda even between epidemics and pandemics.

## Introduction

To improve public health preparedness and response, WHO’s action plan for 2018–2023 aspired for “a European region where the impact of health emergencies is prevented or minimized” [1]. Despite this vision and the growing awareness of the threat of far-reaching pandemics, the extent of the COVID-19 pandemic caught many countries off guard. Although strategic plans based on previous responses existed (like Ebola and Influenza), many countries lacked the readiness to handle a massive-scale infectious outbreak [2–6]. When the COVID-19 pandemic hit Norway in March 2020, the healthcare system did not have unified and detailed organizational plans ready to manage the situation, requiring the development of both national and locally adapted plans [7]. Following democratic self-government, each of Norway’s 356 municipalities is a separate legal entity and may make decisions on its own initiative and responsibility [8]. The municipalities are responsible for providing primary health care services to their inhabitants. Each municipality is obliged to have sufficient community medical and infection control expertise through the employment of one or several Municipal Chief Medical Officers (MCMOs) as a medical advisor(s) [9–11]. Most MCMOs are certified specialists or in training to become specialists in community medicine. Their work areas include environmental health care, public health, mental health care, infection control, emergency preparedness, and medical advice for the municipality’s administrative and political leadership and other municipal service providers [9–13]. The tasks of the MCMO are partly by virtue of provisions in the laws and regulations, and partly laid down in the employment agreement with the municipality [14]. As the municipalities differ substantially in size and organizational structure, the extent of the MCMO resource and the placement in the municipal organization varies. A national survey conducted in September 2020 highlighted both the vulnerability of the MCMO resource and the large differences between the municipalities. While some of the larger municipalities had several MCMOs sharing the role, responsibility and associated tasks, 2/3 of the municipalities only had one MCMO – often with part-time employment and possessing dual roles as GPs or other positions in primary health care [15]. The MCMO has an overarching role in the distinction between clinical medicine and community medicine that requires collaboration across several municipal levels. The fact that the MCMOs are placed at different levels in the organizational structure also seems to affect their professional practice, and several MCMOs have previously reported to have little access to formal decision-making arenas [16].

In Norway, emergency preparedness is regulated by overlapping laws and legislations based on the fundamental principles: responsibility, similarity, proximity and collaboration [17]. The organization in a crisis should remain comparable to the ordinary operation, and at the lowest organizational level possible. The municipal responsibility includes having an emergency plan and a comprehensive risk and vulnerability analysis to ensure adequate local preparedness and collaboration [18]. In a pandemic situation, the Infection Control Act takes effect, and gives comprehensive responsibility and authority to the municipal council for local pandemic management. In urgent situations, the MCMO can exercise the authority of the municipal council [11]. The statutory duty to deal locally with infectious diseases of public concern entails a responsibility for necessary preventive measures, examination, and treatment in primary health care. According to the act, the MCMO is responsible for preparing proposals for emergency/pandemic plans and measures, leading and organizing the pandemic work as well as keeping an overview of the pandemic situation and informing and advising pandemic staff and the municipal population. MCMOs are thus required by law to have a central and responsible role in local pandemic management. At the start of the pandemic, we realized that the crisis would put primary health care to the test. To gain insight into the local response over time and potentially identify take-home lessons to strengthen local future crisis management, we aimed to explore the experiences and views of Norwegian MCMOs on preparedness, collaboration, and municipal crisis organization.

## Materials and methods

### Study design

Longitudinal qualitative interview study with two digital interview rounds [19]. The first interview round took place from September to December 2020, the second round from January to April 2021. Subsequent interviews with a participant were performed 2–4 months apart. This study is part of the CovidNor project. The project consists of several sub studies exploring the management of the COVID-19 pandemic in Norwegian and Swedish primary health care [20].

### Research team

The interdisciplinary research team consisted of five academic/clinical GPs (SHO, SH, AB, PS, IMS), one academic/clinical nurse (LL), one organizational researcher (IS), one general practice academic/clinical otorhinolaryngologist (GH), one professor in general practice (JS), and one professor in medical anthropology (MR).

### Setting, participants and recruitment

The MCMOs were recruited by purposeful sampling, aiming for variation. First, the municipalities were divided into three constructed regions (north, middle, and south of Norway). Then, the municipalities in each region were grouped after population size (small < 10,000 inhabitants, medium 10,000–50,000 inhabitants, large > 50,000 inhabitants) and spread of COVID-19. Based on this categorization, we decided upon which municipalities to approach. The MCMOs were contacted by the research team by email and/or telephone. We initially aimed for ten MCMO interviews in the first round and to re-interview five MCMOs, a pragmatic choice based on feasibility of the study and resources available. After the ninth interview, our preliminary analytical discussions did not reveal any new major insights, and recruitment was stopped. All MCMOs signed informed consents. Participant characteristics are presented in Table 1.

**Table 1.** Characteristics of participating municipal Chief medical Officers (MCMOs).

Participant characteristics (total *n* = 9)	Sex	Age (years)	Experience	MCMO position (%)	Part time employment
Interview 1 (*n* = 9)	5 female, 4 male	38–70 (median 46)	6–20 years (*n* = 8) ≥ 21 years (*n* = 1)	20–100	3 general practitioners (GP)^a^ 1 other part time position
Interview 2 (*n* = 5)	2 female, 3 male	38–70 (median 48)	6–20 years (*n* = 4) ≥21 years (*n* = 1)	20–100	2 general practitioners (GP)^a^ 1 other part time position

^a^ Some of the MCMOs did not have a full-time position as MCMO and worked part-time as GPs or in other part-time position.

### Data collection and analysis

A thematic interview guide (covering preparedness, information/communication, organization, and collaboration) was sent to reference MCMOs for review and adjusted accordingly. Only minor changes were made to the guide after performing one pilot interview, and the interview was included in the study. The interviews were conducted through video meetings by SHO and MR. A written summary was shared with the research team after each interview. After completion of the first interview round, experiences were discussed within the research group to develop a guide for the follow-up interviews. The second interview guide contained the same main themes as the initial guide. However, to pursue personal reflections and themes from the first interview, the guide was personalized for each participant. The interviews were transcribed by two research assistants, and SHO proofread the transcripts while re-listening to all audiotapes. SHO and LL did the initial coding using NVIVO software, and all authors participated in the following analytic process and write up of the paper. We used thematic analysis to analyze the data [21]. Study examples of the methodical steps of Braun and Clarke’s thematic analysis are provided in Table 2. This study was reported in accordance with Standards for Reporting Qualitative Research (SRQR) [22].

**Table 2.** Braun and Clarke’s six stages of thematic analysis with study example.

Phase		Description of analysis step	Example of analysis
1	Familiarizing with the data	Reading and re-reading all transcripts searching for meanings and patterns.	Initial reflections: The MCMOs talked about existing pandemic plans and preparedness. There were differences in how they viewed the preparedness and the existing plans. There were also differences in the organization of the municipality regarding infection control measures, resources, and personnel.
2	Generating initial codes	Organizing the data into meaningful code groups	Preliminary code groups: Barriers to pandemic response Facilitators to pandemic response Sub-groups within each code group: Pandemic plans Equipment Resources and organization of personnel COVID-19 testing Vaccination Future management
3	Searching for themes	Re-focusing the analysis to the broader aspect of themes. Sorting the initial codes into potential and overarching themes.	Theme: Preparedness and pandemic plans Sub-themes: Preparedness/ plans Equipment/testing Future management of the COVID-19 pandemic
4	Reviewing themes	Refinement of candidate themes at the level of the coded data extracts and in relation to the whole data set.	Theme: Pandemic preparedness- from dormant to permanent measure?
5	Defining and naming themes	Identifying the essence of what each team is about (as well as the themes overall) and determine what aspect of the data each team captures as well as naming the themes.	The essence of the theme: A change in the view of preparedness from dormant and de-prioritized to a wish for permanently increased measures.
6	Producing the report	Final analysis and write-up of the report. The analytic narrative needs to go beyond the description of the data and make an argument in relation to the research question.	

## Results

Through the analysis, three major themes were identified; 1) The view of preparedness changed from being low-priority and dormant to the desire to strengthen preparedness as a permanent measure; 2) The nature of the pandemic forced a change in internal and external communication and collaboration for the MCMOs; 3) The pandemic changed the role and position of the MCMO within the municipal organization.

### Pandemic preparedness – from dormant to permanent measure

The MCMOs experienced that preparedness had been de-prioritized and often gave way to more urgent issues pre-pandemic. All the MCMOs expressed a lack of updated pandemic plans, but they had different views on how they perceived preparedness in the initial stages of the pandemic. Some viewed the existing plans as sufficient groundwork for further development and adaption to the current situation.

*«A plan is a good starting point for joint improvisation”. MCMO02*

Having a plan of some kind, no matter how flawed, seemed to speak to an optimistic and action-oriented attitude. These MCMOs reported that the preparedness and pre-existing plans were as good as they could get, given that no one could have predicted the course and extent of the pandemic. However, all MCMOs had to engage in improvised problem-solving because of the outdated plans, and this led some to conclude that the preparedness was insufficient. Limited access to personal protection equipment (PPE) and resources at the start of the pandemic was also challenging for many. At the first interview, all the municipalities in question had their infection detection teams, COVID advisory telephone services and respiratory/COVID clinics up and running.

*“It [the preparedness] is insufficient because we do not have enough resources to do everything else that is important. But it is more correct to say that we have redeployed so that we have sufficient resources.” MCMO03*

The MCMOs described how staffing of new tasks and roles was demanding since the personnel had to be redeployed from other assignments. Although feeling somewhat in control of the situation, the MCMOs made a point of not resting on their laurels due to the still unknown nature of the virus. They emphasized that subsequent outbreaks might play out differently and described how the municipalities had to change their organization of pandemic management. In this regard, they highlighted the importance of prioritizing resources to revise their pandemic plans, quickly re-organize, and take action.

*“We have to make sure we are prepared for anything lying ahead,” MCMO06*

Although most MCMOs perceived the preparedness after the first COVID-wave as sufficient, many pointed out a need for more formalized responsibilities and roles in the municipal response. Several MCMOs postulated the need for long-term planning to keep knowledge and preparedness up to date. They also emphasized the need for larger storages of PPE in the future, both locally and nationally, to become less dependent on import. In the follow-up interviews, vulnerability was still an issue in terms of available resources.

*“It was important to make functions less dependent on people and less vulnerable. We now have four very competent, experienced people who can do the same job.” MCMO04 FU*

Even if the MCMOs at that point had functioning routines in place, scarce personnel resources were still challenging for many. A worry was that people were getting tired of constant preparedness and pandemic work, and several MCMOs expressed a fear of burn-out among staff as well as action fatigue in the population. On the positive side, it seemed like many of the functions within the municipal organization were less person-dependent one year into the pandemic. The fact that several people could fill the roles was reported to spare important strategic personnel and decrease the vulnerability in case of absence due to sick leave or quarantine.

Collectively, the MCMOs in this study drew attention to several challenges and shortcomings of the municipal COVID-19 management. Even though improvements were made through the pandemic, most MCMOs expressed a wish to continue prioritizing preparedness as a permanent measure. More focus on self-containment and to strengthen decentralized competence with separate municipal epidemiological units were launched as ideal solutions to improve future municipal preparedness. Nonetheless, they noted that continuous focus on preparedness would require provision of additional funding for each municipality to be able to carry out all their statutory duties.

### Problem-solving through dialogue, teamwork, and digital networking

The responsibility to manage the information flow, organize and lead the pandemic work fell to the MCMO. In a chaotic crisis, it was challenging to keep up with the extensive and rapidly changing information, and the pandemic forced new strategies for communication and collaboration. The MCMOs were responsible for implementing the information from the National Institute of Public Health (NIPH), the Directorate of Health and the government. Despite being impressed with the information from the NIPH, they experienced the large information flow at the start of the pandemic as demanding.

*“I understand that, on the knowledge side, the paths are made by walking, and that test criteria and recommendations change. But I do not understand the way this has been communicated.” MCMO01*

It was perceived as labor-intensive to keep up to date as information came from various actors on different platforms, sometimes burying important information in the pile of inquiries. Most MCMOs used the webpages from NIPH and Directorate of Health and national press conferences to stay updated, although several pointed out that it initially was difficult to navigate the pages. Grey areas emerged when trying to apply the regulations in clinical practice and to area-specific challenges. Changes in official recommendations were often made without notice, and information was sometimes incoherent. Albeit acknowledging the dynamic situation, several MCMOs found it frustrating to be informed about major changes at the same time as the public *via* national press conferences. Changes in regulations had major ripple effects in the municipalities. The press conferences triggered a flood of inquiries before the municipality had a chance to interpret the changes to local settings, and thereby challenged their ability to keep up with the changes. As the pandemic progressed, the MCMOs described a shift in the communication strategy from central authorities towards engaging in dialogue with the MCMOs. Even though still an overabundance of information, several experienced that the information from the NIPH gradually became more precise, and that an improved dialogue with NIPH had facilitated revision and improvement of the guidelines.

*“They (NIPH) are eager to learn from us in the municipalities. It’s like being an intern and having the best consultant in the world.” MCMO03*

Consensus with the NIPH through dialogue reduced the workload and the level of uncertainty among the MCMOs. At the time of the follow-up interviews, several MCMOs also experienced that their need to be ahead of the public on major changes was met.

Despite continuous challenges in applying the information, most MCMOs experienced to have a good overview of the knowledge base and a strategy on how to organize the pandemic work. Their overall experience was that one-way communication distorted the pandemic work due to uncertainty and misinterpretations while direct dialogue with the opportunity for discussion, knowledge exchange and feedback promoted both internal and external collaboration.

Social distancing and infection prevention considerations quickly shifted communication and collaboration towards digital platforms. The MCMOs used different digital fora to communicate in teams; with the inhabitants, the GPs, leaders of out-of-hours services and municipal health institutions, schools/kindergartens/local businesses, within the municipality organization, local hospitals, neighboring municipalities, a national network of MCMOs, the County Governor and central authorities.

*“We could not use the ordinary organization; we had to establish cross-sectoral groups. We had to make a new organizational map for preparedness.” MCMO07*

Working in teams seemed to enhance the collaboration within the municipality. Teamwork with several MCMOs and/or other medical professionals in the municipality offered support and shared responsibility. Some teams from the 2009 swine flu pandemic were revived. Drawing on previous experiences was described as an advantage enabling the municipality to act more quickly in the initial pandemic wave. The digital availability also permitted the MCMOs to work in teams outside their municipality. Several described an extensive collaboration with other MCMOs through online fora. On this platform, they could discuss and share information, experiences, and routines – and gather questions for the NIPH on behalf of the group. Some experienced massive pressure from central authorities and business organizations to avoid certain local measures, for example, the introduction of quarantine on arrival in the municipality for people who had stayed in other areas of Norway with a high infection rate. In such difficult situations, they found support in the County Governor and the online fora to navigate the information flow as well as to confirm their own understanding of pandemic management. Overall, making new contacts, establishing new arenas for collaboration internally and externally, and getting a better overview of the municipal organization were highlighted as crucial for the pandemic collaboration. Drawing on each other’s expertise and supporting each other in various teams made the MCMOs more confident in their assessments, especially where the knowledge was incomplete, the guidelines unclear/contradictory, or when improvised local solutions were needed.

### The golden age of the MCMOs – brighter future prospects?

Several MCMOs expressed that collaboration with the municipal administrative and political leadership was characterized by a proactive attitude, good interaction and mutual respect for each other’s roles and responsibilities. The majority quickly gained a prominent role in pandemic management as leading medical advisors in the municipality. As a contrast to the positive collaborative experiences, a few reported not to be fully included in the decision-making.

*“I don’t really have any leadership in this pandemic (…) I have had little involvement in the planning of any of this. People given responsibility by someone above me manage this. Sometimes they ask me, but I am generally not involved in the planning process. Things just happen, over my head. I learn about it by chance, in passing.” MCMO08*

Two MCMOs experienced challenges related to exercising their intended role. One was not included in the municipal crisis management group at all, resulting in a feeling of having no authority nor overview over the response and thus not being given the opportunity to fulfill the role of medical advisor. Another MCMO experienced having a position far from where strategic decisions were made, but with great responsibility for the operative pandemic effort in the municipality. This created a mismatch between perceived responsibility and influence on responses with limited opportunity to give feedback and advice. Large size of the municipality and complexity of the municipal organization were factors described to contribute to this de-authorization of the MCMO role.

When the pandemic was ongoing, several MCMOs described that it was simply a matter of rolling up their sleeves and putting other work aside. Many pointed out that they either did not have infection control in their portfolio or that this area constituted a limited part of their everyday work before the pandemic. Most experienced to step out of a secluded role to fill an operational leadership role for a large organization. The pandemic gave a long-awaited boost to MCMOs and the profession of community medicine, and it was described as meaningful and valuable to finally be able to utilize more of their expertise. This change in role also led to increased visibility in the municipality and in the media. The MCMOs were divided as to whether they were comfortable with having a more exposed role, but all emphasized the importance of being visible to create trust and reduce uncertainty in the population. Expectations of increased availability were also a challenge. A majority described how their position was not scaled to handle the massive increase in workload.

*“Overall, I think we have managed, but it has been very overwhelming at times. Extremely overwhelming. Periods of several weeks where you just swim under water.” MCMO05*

Several MCMOs disclosed how the sum of demands blurred the lines between work and private life in the first phases of the pandemic. Increased stress, and especially management of the information overload, was challenging for many, and they felt a heavy responsibility to make interventional decisions based on perceived uncertain professional grounding. Some indicated that they at times were on the verge of what they could endure. One MCMO described that for longer time periods the home was only a place to sleep, while another pointed out that the “parent of the year award” would probably go to someone else. The vulnerability of being alone in the MCMO role was also problematized, highlighting the importance of making the role less person-dependent in the pandemic response. Several coping mechanisms were described; increasing the MCMO resource by adding assistant MCMOs to the team, shielding the MCMO from tasks that could be performed by others or freeing them of combinational roles to focus solely on the pandemic. However, many of these measurements were temporary and guided by the fluctuations of the pandemic, and for some these challenges were still present at the follow-up interviews.

Through close collaboration with the leadership in the municipality, the MCMOs seemed to have gained new insight into the challenges a municipality faces. Likewise, they also experienced that the municipal leaders had realized how their competence could be useful in several new areas. Over the course of the pandemic, the MCMOs had worked actively in formal decision-making arenas and practiced community medicine at a broader scope. This led to reflections on the prospects of their future position within the municipal organization.

*“There is a joke that goes like this: Coming out from the religious service on Christmas Eve, the son asks his mother: “Where do they keep the priest until next Christmas Eve?” Then I have been thinking, “Where do they keep the MCMO until the next pandemic? Do we still get the same opportunities and placement in the organization that enables us to give good advice for the municipality leaders to manage in practice?” MCMO07*

As several experienced “rising through the ranks” and holding a more central position in the municipal organization during the pandemic, many wondered how the role would turn out after the pandemic. The quoted MCMO questioned whether they would be given the same opportunity and impact to perform community medicine within the municipality in the future. The MCMO pointed out that according to basic principles of preparedness, the municipal organization should ideally be reflected both in crisis and in times of peace, calling for an evaluation of the ordinary organization. The MCMO thus emphasized the importance of taking lessons from the organization during the pandemic when developing the future post-pandemic MCMO role.

## Discussion

### Principal findings

Our analysis revealed a change in the MCMO perception of adequate crisis preparedness - from being a low-priority area to a desire to maintain a higher level of preparedness as a permanent measure.

Infection prevention regulations, lack of detailed pandemic plans and the extent of the pandemic forced the MCMOs to change their internal and external communication and collaboration towards direct dialogue, teamwork, and digital networking.

The pandemic changed the role and position of the MCMO within the municipal organization. Although most MCMOs were given a central role as medical advisors and leaders in the municipal pandemic response, some MCMOs experienced that they were not positioned to fully exercise their intended role. In our material, de-authorization of the MCMO role seemed to coincide with increasing size and organizational complexity of the municipality.

### Strengths and limitations

We consider it a strength that the longitudinal design allowed us to follow up on experiences and reflections over time. We also believe it is of value to have access to reflections the MCMOs made while still in the middle of the pandemic crisis.

Digital meetings can be perceived less personal, make interpersonal connections more difficult and thus be regarded a limitation. Contrarywise, the use of digital platforms may have made sharing of personal reflections easier for the MCMOs.

Lacking the perspective of a MCMO in the analytic process may be considered a limitation. However, analyzing without the preconceptions of a MCMO may also have made us more open to the material and allowed us to analyze the data more freely.

Several of the authors are GPs. Going into the project, we were aware that our own preconceptions could make us more prone to interpret the interviews from a GP’s point of view, especially regarding municipal collaboration. When performing the analysis, we tried to keep this in mind, and we believe that being an interdisciplinary team helped us maintain reflexivity through analytical discussions.

Even though data was collected in the middle of the pandemic, the coding and final analysis of the material did not take place until it subsided. This may be seen as a limitation as our knowledge base and situation awareness inevitably were different. Nevertheless, it may also have helped the contextualization and interpretation of the themes as well as drawing the overall lines of the analysis.

### Findings in relation to theory and other studies

The pandemic response in Norway was reported to score high on both an epidemic preparedness index [23], a resilience index (reduction of negative impact of mortality related to COVID-19), and a preparedness/prevention index (COVID-19 vaccination) [24]. Regardless of a successful response when evaluated retrospectively, several MCMOs in our study experienced the pandemic preparedness as insufficient while working through the pandemic, especially in the early phases. This inadequacy was linked to a lack of detailed plans for the distribution of responsibilities and organization of the response, as well as an initial lack of PPE and overall vulnerable personnel resources. According to Kruk et al. “Health system resilience can be defined as the capacity of health actors, institutions, and populations to prepare for and effectively respond to crises; maintain core functions when a crisis hits; and, informed by lessons learned during the crisis, reorganize if conditions require it” [25]. All MCMOs described how the lack of tailored plans forced them to improvise, draw on their own experiences, adapt and reorganize on the go. This ability was highlighted as necessary and important traits of the municipal response and can also be described as collective improvisation [26] and real-time experiential learning [27]. However, to which extent all their core tasks were satisfactorily covered was debated by the MCMOs. Several expressed concerns about prioritizing the pandemic over what they perceived as important ordinary tasks, like mental health care and other aspects of public health. In their experience, preparedness was a low priority measure pre-pandemic due to tight economical frameworks and the demands of the statutory municipal obligations. This resonates with findings from a previous study from our research group, where the MCMOs reported that infection control was considered a significant part of their job. However, due to a lack of capacity, quality improvement work in the field of infection control was often de-prioritized resulting in a case-by case management [28]. The MCMOs experienced that emergency preparedness had to give way in the normal situation. When the focus shifted abruptly to pandemic preparedness, several MCMOs reported that they were occupied with infection prevention and control to an extent where they were not able to fulfill other ordinary obligations. One of our main findings was the change in the view of preparedness among the MCMOs, from a perceived dormant measure to a wish for prioritizing preparedness permanently. The desire to develop and maintain preparedness post-covid to preserve health system resilience is not unique to the Norwegian setting. However, in this ambition lies an implicit precondition of increased funding allocated to public health measures [3,29,30].

Our findings show that the MCMOs initially struggled to keep up with the constant modifications of information and regulations. Similar challenges are also reported in primary health care in other countries [31–34]. The flow of information started out in a top-down manner, with the health authorities providing regulations and instructions. As the pandemic progressed, the MCMOs experienced a shift from one-way communication to a dialogue-based and collaborative approach. In response to infection prevention regulations, the MCMOs shifted the mode of communication and arena for collaboration to different digital fora. This re-organization gave the MCMOs the opportunity to collaborate in teams with various actors within and across municipalities. Such teams were by one MCMO referred to as “dream teams”. These formal and informal networks were perceived highly useful to coordinate the response and to align national measures with local needs and challenges, also noted by other Norwegian studies [35,36]. In this way, the MCMOs and their numerous collaborators made sense of the situation together and reached consensus on how to respond effectively through sharing of information, interpretations, and perspectives. Broom et al. highlighted leaders’ view on collaboration and sensemaking as critical means to achieve adaptability and resilience [37], while Karreinen et al. identified teamwork as a key protective factor to reduce stress among heath care personnel [38]. Weick and Sutcliffe’s sensemaking theory [39,40] emphasizes that in complex and uncertain situations like crises, individuals within organizations come together to collectively make sense of the situation. Teamwork plays a pivotal role in sensemaking. Crisis situations demand rapid and accurate information sharing, collaborative problem-solving, and joint decision-making. Effective teamwork ensures that different perspectives are considered, diverse expertise is leveraged, and potential blind spots are identified. In accordance with the theory, teamwork can help foster a shared mental model among team members, enhancing their ability to anticipate, adapt, and respond to unexpected events.

The pandemic also changed the role and position of the MCMO within the municipal organization. Fossberg and Frich explored the MCMÒs perception of their own role pre-pandemic and found that many experienced to have little access to decision-making arenas [16]. We found that MCMOs utilized more of their community medicine expertise through the pandemic, and that most of them received a leading role in the municipal pandemic response, also observed by Hungnes et al. [35]. In our material, larger municipalities with more complex organization seemed to coincide with less inclusion of MCMOs in formal decision-making arenas during the pandemic. Although raising interesting questions, this finding was only based on experiences from two informants from larger municipalities, and should be explored by further research. In an editorial, Renaa points out that local authorities should make better use of the MCMOs by familiarizing themselves with what their local public health expertise can bring to planning and running day-to-day municipal operations [41]. All though several MCMOs in our study experienced that municipal leaders during the pandemic had realized that their competence could be useful in several new areas, they also questioned their future role and position within the municipal hierarchy. One MCMO in our study even pointed to the basic principles for security and civil protection stating that the organization during a crisis should remain similar to the ordinary organization. Following that logic, the MCMO should remain in the same (higher) level of the municipal organization post-pandemic. According to a commentary by Raastad, it is a mutual responsibility between the municipality and each MCMO to engage in dialogue about what is needed to fulfill tasks and obligations as well as how to make best use of the competence of the MCMO [14]. In other words, the placement in the municipal organization and the definition of the MCMO role seems to rely on the interplay between the local authorities and the MCMOs themselves. In a recently published study exploring the post-pandemic MCMO role, Haugestuen and Feiring concluded that "the pandemic effect” seemed to be over, leaving the MCMOs yet again to fight for their place at important decision-making arenas [42].

### Implications for practice, policies, and research

We found that the MCMOs wanted to prioritize preparedness post-pandemic and keep their place in decision-making arenas. When the pandemic boost ceases, it seems to be up to each MCMO to engage in dialogue with local authorities to maintain their position in the municipal hierarchy. Standardizing the MCMO placement within the municipal structure is one way to resolve this issue. The question is whether this is feasible given the great variation in available MCMO resources, size, and organizational complexity between the Norwegian municipalities. As highlighted by the MCMOs, the municipal organizational and financial framework did not leave room to focus on preparedness before the pandemic. Thus, additional financial resources designated to public health are a precondition for maintaining health system resilience and prioritize preparedness as a permanent measure.

## Conclusions

The Norwegian pandemic response and outcome have been regarded as successful internationally. Although the MCMOs managed to implement flexible and quick responses facilitated by teamwork, dialogue and joint sensemaking, they also identified several challenges and shortcomings of the Norwegian pandemic preparedness requiring organizational and financial changes to sustain future health system resilience.
